# Recombination Form and Epidemiology of HIV-1 Unique Recombinant Strains Identified in Yunnan, China

**DOI:** 10.1371/journal.pone.0046777

**Published:** 2012-10-09

**Authors:** Lin Li, Lili Chen, Shaomin Yang, Tianyi Li, Jianjian Li, Yongjian Liu, Lei Jia, Bihui Yang, Zuoyi Bao, Hanping Li, Xiaolin Wang, Daomin Zhuang, Siyang Liu, Jingyun Li

**Affiliations:** 1 Department of AIDS Research, State Key Laboratory of Pathogen and Biosecurity, Beijing Institute of Microbiology and Epidemiology, Beijing, China; 2 Yunnan Provincial Hospital Infectious Disease, AIDS Care Center (YNACC), Kunming, Yunnan, China; 3 Urumqi General Hospital of Lanzhou Military Area Command, Urumqi, Xinjiang, China; Institute of Infectious Disease and Molecular Medicine, South Africa

## Abstract

Several studies identified HIV-1 recombination in some distinct areas in Yunnan, China. However, no comprehensive studies had been fulfilled in the whole province up to now. To illustrate the epidemiology and recombination form of Unique Recombinant Forms (URFs) circulating in Yunnan, 788 HIV-1 positive individuals residing in 15 prefectures of Yunnan were randomly enrolled into the study. Full-length *gag* and *pol* genes were amplified and sequenced. Maximum likelihood tree was constructed for phylogenetic analysis. Recombinant breakpoints and genomic schematics were identified with online software jpHMM. 63 (10.2%) unique recombinant strains were identified from 617 strains with subtypes. The URFs distributed significantly differently among prefectures (Pearson chi-square test, P<0.05). IDUs contained more URFs than sexual transmitted population (Pearson chi-square test, P<0.05). Two main recombinant forms were identified by considering the presence of CRF01_AE segments in full length *gag*-*pol* genes, which were B′/C and B′/C/CRF01-AE recombinants. Three clusters were identified in the ML tree which contained more than three sequences and supported by high bootstrap values. One CRF was identified. Many of URFs contained identical breakpoints. The results will contribute to our understanding on HIV recombination and provide clues to the identification of potential CRFs in China.

## Introduction

Human immunodeficiency viruses (HIV) were characterized with high level of genetic variation. Recombination is one of main mechanisms of HIV diversity. When a cell that was dually infected by two different viruses produced progeny virions with genomic RNAs from each virus, strand-switching would take place during the next round of reverse transcription [1], [2], [3]. As the results, recombination would happen and unique variants with genome from two distinct parental viruses would be produced. Substantial studies showed that recombination contributed to viral escaping mutations under immune pressure, viral fitness and emergence of viral drug-resistance [4]. Up to now, 52 circulating recombinant forms (CRFs) (http://www.hiv.lanl.govcontenthiv-dbCRFsCRFs.html) have been reported, which is responsible for more than 20% HIV infections in the global AIDS epidemic [5]. Many studies demonstrated that mosaic strains were arising frequently, especially in populations with multiple subtypes circulating [6], surveillance on the emergence and epidemic of HIV-1 unique recombinant forms will be helpful for the prediction of CRFs and HIV epidemic in different area.

Yunnan, a southwestern province of China that shares a border with the famous heroin-producing area of Myanmar (Burma), was considered as the epicenter of China. The first HIV-1 epidemic that occurred among injecting drug users (IDUs) in 1989 in China was reported inYunnan [7], [8], [9]. Now it is still the area with the most severely HIV epidemic in China with highest number of new reported cases. Multiple HIV-1 genotypes, including B, C, CRF01_AE, CRF07_BC, and CRF08_BC, are all currently circulating in the area [10], supposing the possible emergence of a new recombination. CRF07_BC and CRF08_BC, the dominant CRF strains spreading in China now, were believed to arose in Yunnan in 1990s and spread to other areas [11], [12]. In recent years, the transmitting routes and prevalence of different HIV subtypes or CRFs have changed dramatically in Yunnan [13]. Accordingly, URFs originated from prevalent subtypes in same population would change. Recently, extensive recombination of HIV was identified in Myanmar, which is close to Yunnan province [14]. All of these reasons urgent the surveillance of HIV recombination in Yunnan, China. In this study, we characterized the genomic schematic and illustrated the epidemic of HIV URFs in whole Yunnan province basing on full length *gag*-*pol* gene sequences.

## Materials and Methods

### Study subjects and specimens

HIV-positive individuals were randomly recruited from the list of Yunnan AIDS care center, with written informed consents. The ratios of enrolled subjects from different prefectures were determined according to the reported cases of prefectures to make them representatively. The epidemiologic background was collected through specific epidemiological investigation by trained interviewers. Peripherial blood was sampled from Jan. 2008 to Dec. 2009, and plasma was separated and stored in −80°C freezer. The Ethical Review Board, Science and Technology Supervisory Committee at the Beijing Institute of Microbiology and Epidemiology approved the study.

### HIV-1 RNA extraction, amplification and sequencing

Viral RNA was extracted from 500 µl HIV-1 positive plasma specimens with high pure viral RNA kit (Roche, USA). Viral full length *gag* (from 763 to 2400 according to the HXB2 calibrator) and *pol* (from 2068 to 5221 according to the HXB2 calibrator) genes were amplified separately with more than 300 bp overlapping region using reverse transcriptional nest PCR as described before [15]. Positive PCR products were sequenced by Huada genomics company (China) with a variety of internal specific primers (available on request) after being purified. For variant containing breakpoint in the overlapping regions of *gag* and *pol* genes, 5′ half genome was amplified and sequenced again using methods provide in previous paper [16]. The nearly full-length genomes were reverse-transcripted and amplified in two halves with 1 kb overlapping regions as described before [16].

### Edit, Assemble, Genotyping and Phylogenetic Analysis of HIV-1 Sequences

All of the sequenced fragments were edited and assembled as described before [16]. To check for potential contamination, the sequences obtained were compared to all known sequences in the HIV database by a Basic Local Alignment Search Tool (BLAST) search (http://hiv-web.lanl.gov/content/index). HIV genotype was determined using the national center for biotechnology information viral genotyping tool (http://www.ncbi.nih.gov/projects/genotyping/formpage.cgi) by combining both gene regions from same isolate and further confirmed by phylogenetic analysis with reference sequences using the Neighbor-Joining method in MEGA5.0 software and Maximum Likelihood method in PhyML software [17]. To fulfill the phylogenetic analysis, full length *gag* and *pol* genes derived from same variant were assembled together and aligned with reference sequences representing subtypes A–D, F–H, J, K, CRF01_AE, CRF07_BC, CRF08_BC and CRF31_BC (http://www.hiv.lanl.gov). ML tree was constructed using GTR plus gamma model of nucleotide substitution which was selected with jModelTest software [17], [18]. SPR was used for tree searching. 100 bootstrap replicates were fulfilled to decide the branch support value. The possible intertype mosaicism was screened with online Recombination Identification Program (version 3.0; http://hiv-web.lanl.gov) and further confirmed by the online software jpHMM-HIV (http://jphmm.gobics.de/). The breakpoints were determined by a probabilistic approach using jpHMM-HIV online software [19]. The approach combines the idea of a profile HMM with the jumping alignment (JALI) approach proposed by Spang et al. in 2002 as a strategy to database searching.

### Statistical analysis

Statistical analyses were performed using SPSS V.10.0 software. Pearson chi-square test was used to analyze the differences of demographic distributions of newly identified URFs and difference s of composition of URFs' genome. All statistical analyses were two sided, and p<0.05 was considered statistically significant.

## Results

### Demographic distributions of identified URFs

A total of 788 participants residing in Yunnan province were enrolled into the study. Among of them, 617 subjects were determined with infected viral subtypes. 63 strains (accession number: JQ898162- JQ898277, JX679207), which were 10.2% of subtype-determined isolates, showed different genomic schematic structure to reported CRFs and were identified as URFs. The prevalence of URFs was unequal in different prefectures in Yunnan province (Table 1, Pearson chi-square test, P<0.05). Prevalence of URFs in Dehong and Dali prefectures were higher than 20%, which strongly suggested that there was high prevalence of more than one subtype in the same population. Relative low prevalence of URFs in Xishuangbanna and Lincang prefectures were observed, which suggested relatively sole subtype of HIV prevalent in the area. Epidemic of URFs in peoples acquired HIV-1 through different transmitting routes also largely varied (Pearson chi-square test, P<0.05). Among 129 strains from IDUs, 20 URFs were identified (15.5%). In heterosexual transmitted population, the prevalence of URFs was only 7.9%.

**Table 1:** Distribution of URFs in different prefectures and transmitting routes in Yunnan.

Prefectures and transmitting routes		URFs	Cases with subtypes	Prevalence (%)
Prefectures	Dehong	29	94	30.9
	Dali	5	19	26.3
	Kunming	9	61	14.8
	Zhaotong	2	15	13.3
	Puer	4	38	10.5
	Honghe	4	41	9.8
	Baoshan	2	21	9.5
	Xishuangbannan	2	91	2.2
	Lincang	3	186	1.6
	Other prefectures	0	25	0.0
	Unknown	3	26	11.5
Transmitting routes	Sexual transmission	32	407	7.9
	IDUs	20	129	15.5
	Others	11	81	14.3
Total		63	617	10.2

### Genomic schematic structure analysis

Since CRF01_AE, CRF07_BC, CRF08_BC and subtype B′ were the main prevalent strains in Yunnan province, it is not surprising for us to find that all genomes of the URFs were comprised with them (Table 2). Strains composed of subtype B′ and C in the *gag*-*pol* regions (47 cases) were dominant in the URFs, which were responsible for 74.6% of total URFs. Subtype B′ and C were believed to be introduced into Yunnan province and responsible for early HIV epidemic, especially in the IDUs [20], [21]. CRF01_AE was found to lead HIV epidemic in heterosexual transmitted population in recent years [13]. That may be the reason why most of URFs with CRF01_AE as genomic backbones were found in heterosexual transmitted population (75%). The distribution of URFs with different backbones was significantly different in IDUs and heterosexual transmitted population. Comparing to the data published by Yang et al in 2002, the composition of *gag*-*pol* genes also changed. The ratios of HIV-1 variants containing CRF01_AE segment in *gag*-*pol* genes increased from 8.3% in 2002 in Yang's paper to 25.4% in 2009 in this manuscript, which strongly suggested that the recombination form of URFs might be changing.

**Table 2:** Distribution of URFs of different patterns in transmitting routes.

Patterns of URFs	Transmitting routes			Total
	Sexual transmission	IDUs	Others	
B′/C	20(31.7%)	19(30.2%)	8(12.7%)	47(74.6%)
B′/C/CRF01_AE	12(19.0%)	1(1.6%)	3(4.8%)	16(25.4%)
Total	32(50.8%)	20(31.7%)	11(17.5%)	63(100%)

### Phylogenetic analysis of URFs

**Figure 1:**
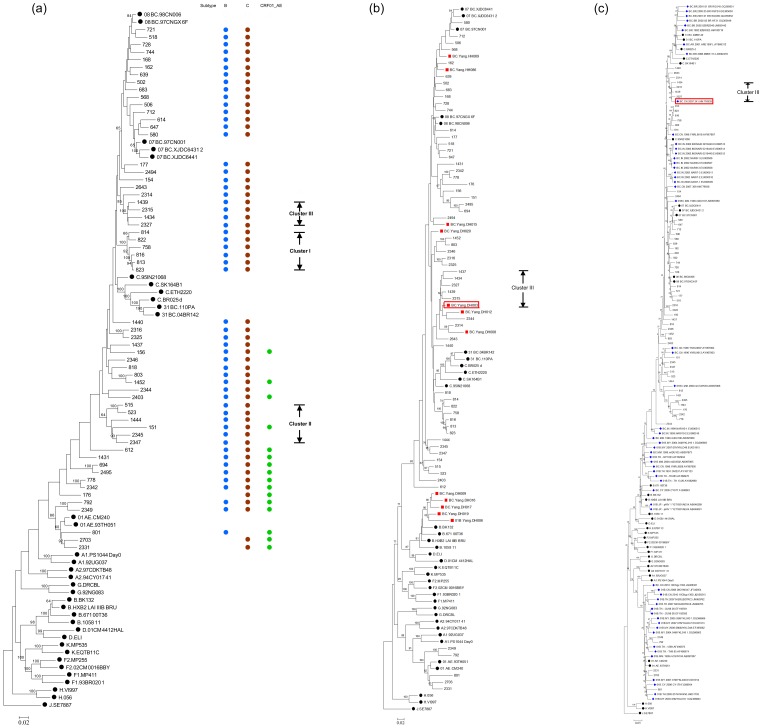
Phylogenetic tree analysis. (a) Full length *gag* and *pol* genes from same isolates were assembled together. Maximum Likelihood trees created with the 59 full length *gag*-*pol* gene of HIV-1 URFs sequences from Yunnan and a selection of reference sequences of subtype A–D, F–H, J, K, CRF01_AE, CRF07_BC, CRF08_BC and CRF31_BC (black dots, http://hiv-web.lanl.gov/- see Materials and Methods for details of selection of reference sequences). The length of sequences used in the analysis was 4307 base pairs respectively by using HXB2 as the reference genomic. Each reference sequence is labeled with the HIV-1 subtype, followed by name. Branch support values greater than 60% are indicated at the corresponding nodes of the tree. The scale bar represents 2% genetic distance. The compositions of genomes were indicated in right column. Three clusters containing more than 3 strains with high branch support values were labeled accordingly. (b) Full length *gag* and partial *pol* genes (2.6 Kb) of 12 strains from Yang's paper were aligned together with 59 strains identified in this study and subtype references sequences. ML tree was constructed as described in the methods. Strains from Yang's paper were labeled with the HIV-1 subtype, followed by “Yang” and strain's name. Branch support values greater than 60% are indicated at the corresponding nodes of the tree. All strains from Yang's paper were labeled with red square (▪). Strain located in red rectangle was the one (DH003) presenting same genomic structure as strains in Cluster III. (c) Full length *gag*-*pol* genes downloaded from HIV Los Alomas database of URFs in Asia containing B, C or CRF01_AE segments were aligned with strains identified in this study. ML tree was constructed as described in the methods. Strains downloaded from database were labeled with the HIV-1 subtype, followed by country, year, name and accession number. Branch support values greater than 60% are indicated at the corresponding nodes of the tree. All strains from database were labeled with blue diamond (♦). Strain located in red rectangle was the one (341) presenting same genomic structure as strains in Cluster III.

In the maximum likelihood tree constructed with full-length *gag*-*pol* genes sequenced in this study, all of unique recombinant strains placed among the subtype reference sequences, with three clusters containing more than 3 sequences with high branch supports in the ML tree. (Figure 1) With detailed URF sequence data presented by Yang et al in 2002, phylogenetic analysis was further fulfilled to explore possible evolution relationship. To do this, all *gag*-*pol* genes from URFs reported in this study were aligned with 12 URF sequences identified in Yang's paper and ML tree was constructed basing on 2.6 Kb *gag*-RT genes as described in the materials and methods. Similar topology was observed as the ML tree constructed with only our sequences. DH003, DH008 and DH012 from Yang's lab were found locating in Cluster III. To identify more HIV-1 variants with similar recombinant form, all of Asian URFs (57 strains) containing subtype B, C and CRF01_AE gene segments in *gag*-*pol* regions were downloaded from Los Alomas database and analyzed together with our sequences. Phylogenetic tree showed that YNRL9607, YNRL9613 grouped into cluster II. Strain 341 grouped into cluster III.

### Analysis of recombinant breakpoint and identification of a novel CRF

**Figure 2:**
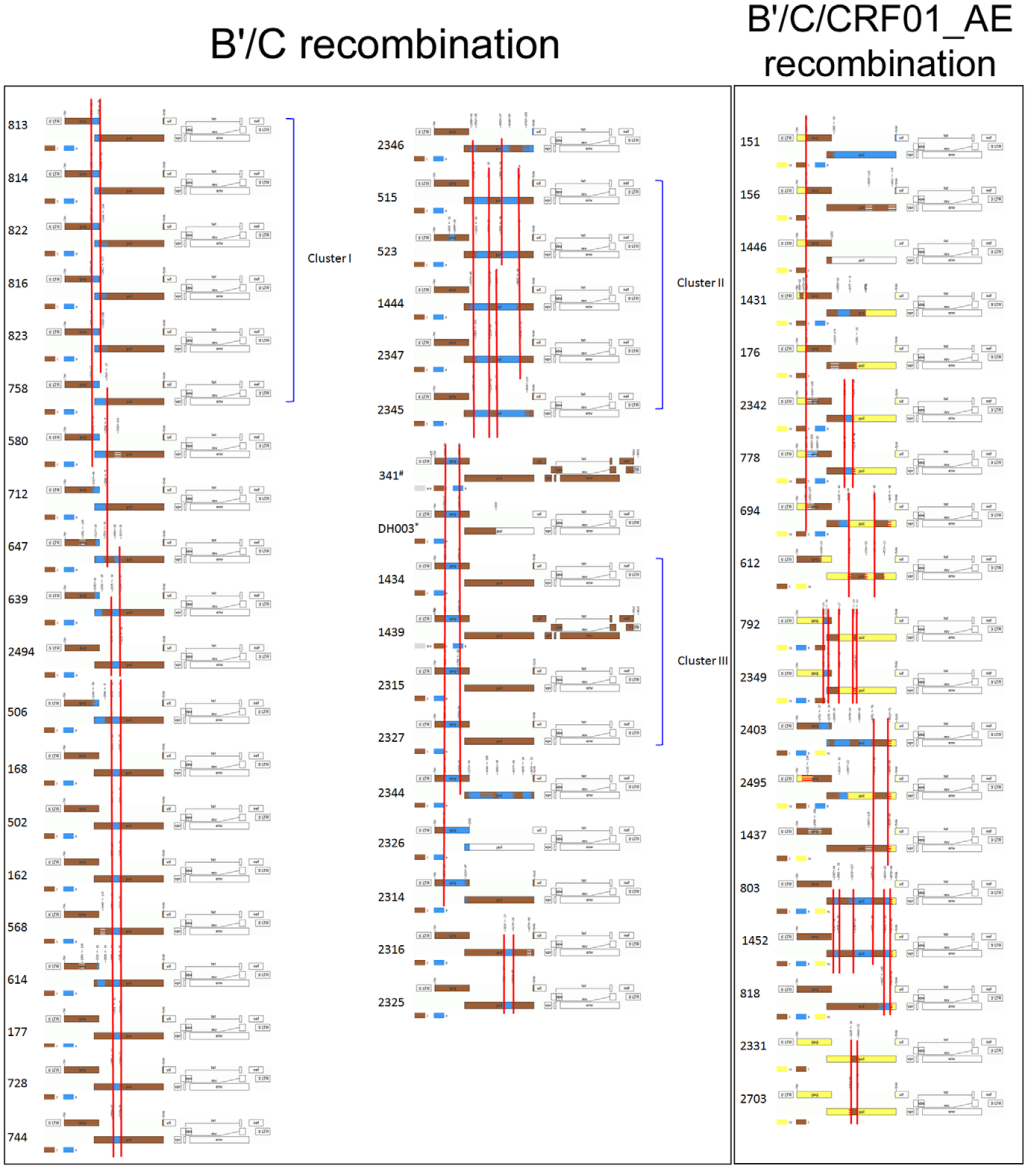
Genome maps of unique recombinant forms harboring same breakpoints. jpHMM-HIV software available at (http://jphmm.gobics.de/) was used to calculate the posterior probabilities of the subtypes at each sequence position and draw the genome map. All genome composed of subtype B′ and C were put in the left frame while genome containing CRF01_AE segments were put in the right frame. Same breakpoints in different genome schematics were labeled with red line. *DH003 strains was obtained from Yang's paper [22]. # 341 strain was obtained from HIV Los Alomas database and published in Liu's paper [26].

Recombinant breakpoints analysis basing on full length *gag*-*pol* sequences showed that 56 of 63 URFs contained same breakpoints with at least one of other strains.(Figure 2) Similar genomic schematic structures among sequences in the same cluster were found. In cluster I, 813 and 814 contained same breakpoints, while 816, 822 and 823 shared same breakpoint. All of those 5 strains were from same area, it is hard to exclude epidemiological linkage. In cluster II, 515 and 523 contained similar breakpoints in *pol* gene, while 523 had a short 230 bp length subtype B′ gene segment inserting in *gag* region. The other sequences locating in cluster II contained longer subtype B′ sequences in *pol* gene. 1444 and 2347 contained same genomic schematic and might be potential CRF. All of sequences locating in cluster III contained same recombinant breakpoints (1229 and 1837 according to HXB2 calculator) with subtype B′ gene segments inserted in *gag* gene. Three strains (DH003, DH012 and DH015) in professor Yang's paper contained same breakpoints as strains in cluster III identified in this study. Epidemiological background investigations excluded linkage among those participants (Table 3) and suggested that those strains might represent a new CRF. All full length *gag*-*pol* sequences of URFs containing B′, C or CRF01_AE segment from Asian were obtained from database and submitted to breakpoints analysis. The results showed that strain 341 had similar breakpoint as 1434, 1439, 2315, 2327 strains, and also the strain DH003 from Yang's paper. To determine the full length genomic schematic, 3′ half genomic sequences of 1439 strain identified in this study were obtained as described in the materials and methods. The jpHMM analysis showed that 1439 and 341 strains displayed same genomic structure (Figure 2). In the chimera, a subtype B′ segment was inserted into the backbone of subtype C, with breakpoints corresponding to HXB2 nucleotide positions 1229 and 1837 approximately. According to the criteria for designation of a new CRF, those 6 strains constitute a new CRF identified in the worldwide HIV-1 pandemic.

**Table 3:** Information on subjects harboring novel B′/C recombinants in Yunnan China.

Patients	Age	Gender	Country	Year of fist positive HIV test	Year of collection	Risk factor	Viral Load	CD4 cell count	Accession number
DH003*	-	-	CN	-	2000–2001	IDU	-	-	AB078705
1434	3	Female	CN	2008	2009	MCT	109361	884	JQ898195, JQ898254
1439	30	Male	CN	2008	2009	IDU	96639	379	JX679207
2315	45	Male	CN	2007	2009	IDU	49795	276	JQ898203, JQ898262
2327	9	Female	CN	2007	2009	MCT	1361	142	JQ898207, JQ898265
341#	35	Male	CN	2007	2009	IDU	17963	319	HM776939

# : 341 strain was downloaded from HIV Los Alomas database which was also published by our lab. [26].

* : DH003 strain was identified by Yang et al. in 2002. [22].

## Discussion

High heterogeneity of HIV was always observed in infected individuals, which made it difficult for the amplification and sequencing of long gene segments. In most of previous studies, the determination of HIV subtypes prevalent in Yunnan province was always based on short gene segment, which could not conclude HIV-1 subtypes and identify recombination exactly. Long gene segment, even full length genomic sequence, is now considered more suitable for determining HIV subtypes. Many studies obtained long gene segment through assembling many short segments, however, it was hard to get gene segment originated from same variants due to the existence of quasispecies. In this study, we analyzed HIV genomic schematic structure basing on full length *gag* (1584 bp according to HXBII calculator) and *pol* (3147 bp according to HXBII calculator) genes, which make it more exactly for identification of recombinant strains.

HIV epidemic in Yunnan province was characterized with multiple strains prevalent simultaneously. The subtype constitution in Yunnan has always been changing. Accordingly, epidemiology of URFs would change. In 2002, Yang R et al. found that URFs accounted for 70% of HIV prevalent in IDUs in Dehong prefecture [22]. In this more comprehensive study basing on long gene segments, highest prevalence of URFs in IDU population was also observed. However, we found that the prevalence of URFs in IDUs in Dehong prefecture was 35% (14/40). Due to different genomic regions were adopted in determining viral subtypes, it is kind of hard to compare both results, but our data supposed the possible change of URFs prevalence in Yunnan. The recombinant forms of URFs were also in changing. In previous studies, URFs containing genomes composed of subtype B′ and C were dominant [22], [23]. In this study, although recombinant strains originated from subtype B and C segments were still dominant in URFs, more URFs with CRF01_AE as the backbone were identified. CRF08_BC strains were dominating HIV epidemic in Yunnan province, which was responsible for more than 50% HIV cases (data not shown). Many URFs containing CRF08_BC breakpoints were identified in this study. So it was necessary to pay attention to URFs originated from CRF08_BC which may become a CRF. CRF01_AE was believed to be introduced into Yunnan heterosexual transmitted population in few years [13] and leading a new epidemic in Yunnan. Identifications of URFs originated from it strongly suggested that the subtype is spreading quickly and need to be put more attention in the surveillance of HIV in the area.

In this study, at least three clusters of sequences with similar recombinant sites were identified. One CRF was identified with sequences obtained from HIV Los Alomas database and Yang's paper [22]. Initially, subtype B′ was believed to be main subtypes in IDUs in Yunnan [20], [21], [24], [25]. However, CRF07_BC and CRF08_BC strains spread quickly after their formation and became dominant in Yunnan and many other areas in China [11]. Comparing to their parental strains, CRF07_BC and CRF08_BC showed more superiority of spreading in Chinese population. Since many new URFs originated from now prevalent strains were observed, there is the possibility of new CRFs displaced them and become dominant. Further studies were necessary to explore the biological characterizations of URFs and social factors related to their epidemic, which will provide predictive information on HIV in Yunnan and even China.
